# Access to medicines by patients of the primary health care in the Brazilian Unified Health System

**DOI:** 10.11606/S1518-8787.2017051007139

**Published:** 2017-09-22

**Authors:** Juliana Álvares, Augusto Afonso Guerra, Vânia Eloisa de Araújo, Alessandra Maciel Almeida, Carolina Zampirolli Dias, Bruna de Oliveira Ascef, Ediná Alves Costa, Ione Aquemi Guibu, Orlando Mario Soeiro, Silvana Nair Leite, Margô Gomes de Oliveira Karnikowski, Karen Sarmento Costa, Francisco de Assis Acurcio

**Affiliations:** I Departamento de Farmácia Social. Faculdade de Farmácia. Universidade Federal de Minas Gerais. Belo Horizonte, MG, Brasil; II Pontifícia Universidade Católica de Minas Gerais. Belo Horizonte, MG, Brasil; III Faculdade de Ciências Médicas de Minas Gerais. Belo Horizonte, MG, Brasil; IV Programa de Pós-Graduação em Medicamentos e Assistência Farmacêutica. Faculdade de Farmácia. Universidade Federal de Minas Gerais. Belo Horizonte, MG, Brasil; V Programa de Pós-Graduação em Saúde Pública. Faculdade de Medicina. Universidade Federal de Minas Gerais. Belo Horizonte, MG, Brasil; VI Instituto de Saúde Coletiva. Universidade Federal da Bahia. Salvador, BA, Brasil; VII Faculdade de Ciências Médicas. Santa Casa de São Paulo. São Paulo, SP, Brasil; VIII Faculdade de Ciências Farmacêuticas. Pontifícia Universidade Católica de Campinas. Campinas, SP, Brasil; IX Departamento de Ciências Farmacêuticas. Universidade Federal de Santa Catarina. Florianópolis, SC, Brasil; X Faculdade de Ceilândia. Universidade de Brasília. Brasília, DF, Brasil; XI Núcleo de Estudos de Políticas Públicas. Universidade Estadual de Campinas. Campinas, SP, Brasil; XII Programa de Pós-Graduação em Saúde Coletiva. Departamento de Saúde Coletiva. Faculdade de Ciências Médicas. Universidade Estadual de Campinas. Campinas, SP, Brasil; XIII Programa de Pós-Graduação em Epidemiologia. Faculdade de Medicina. Universidade Federal do Rio Grande do Sul. Porto Alegre, RS, Brasil

**Keywords:** Pharmaceutical Services, Health Services Accessibility, Primary Health Care, Health Services Research, Brazilian Unified Health System, Assistência Farmacêutica, Acesso aos Serviços de Saúde, Atenção Primária à Saúde, Pesquisa sobre Serviços de Saúde, Sistema Único de Saúde

## Abstract

**OBJECTIVE:**

To evaluate the access to medicines in primary health care of the Brazilian Unified Health System (SUS), from the patients’ perspective.

**METHODS:**

This is a cross-sectional study that used data from the *Pesquisa Nacional sobre Acesso, Utilização e Promoção do Uso Racional de Medicamentos* – Services, 2015 (PNAUM – National Survey on Access, Use and Promotion of Rational Use of Medicines), conducted by interviews with 8,591 patients in cities of the five regions of Brazil. Evaluation of access to medicines used concepts proposed by Penshansky and Thomas (1981), according to the dimensions: availability, accessibility, accommodation, acceptability, and affordability. Each dimension was evaluated by its own indicators.

**RESULTS:**

For the “availability” dimension, 59.8% of patients reported having full access to medicines, without significant difference between regions. For “accessibility,” 60% of patients declared that the basic health unit (UBS) was not far from their house, 83% said it was very easy/easy to get to the UBS, and most patients reported that they go walking (64.5%). For “accommodation,” UBS was evaluated as very good/good for the items “comfort” (74.2%) and “cleanliness” (90.9%), and 70.8% of patients reported that they do not wait to receive their medicines, although the average waiting time was 32.9 minutes. For “acceptability,” 93.1% of patients reported to be served with respect and courtesy by the staff of the dispensing units and 90.5% declared that the units’ service was very good/good. For “affordability,” 13% of patients reported not being able to buy something important to cover expenses with health problems, and 41.8% of participants pointed out the expense with medicines.

**CONCLUSIONS:**

Results show 70%–90% compliance, which is compatible with developed countries. However, access to medicines remains a challenge, because it is still heavily compromised by the low availability of essential medicines in public health units, showing that it does not occur universally, equally, and decisively to the population.

## RESUMO

**OBJETIVO:**

Avaliar o acesso aos medicamentos na Atenção Primária em Saúde do Sistema Único de Saúde na perspectiva do usuário.

**MÉTODOS:**

Estudo transversal que utilizou dados da Pesquisa Nacional sobre Acesso, Utilização e Promoção do Uso Racional no Brasil – Serviços, 2015, realizado por meio de entrevistas com 8.591 usuários em municípios das cinco regiões do Brasil. A avaliação do acesso aos medicamentos utilizou conceitos propostos por Penshansky e Thomas (1981), segundo as dimensões: disponibilidade, acessibilidade geográfica, adequação, aceitabilidade e capacidade aquisitiva. Cada uma das dimensões foi avaliada por meio de indicadores próprios.

**RESULTADOS:**

Para dimensão disponibilidade, 59,8% dos usuários declararam ter acesso total aos medicamentos, sem diferença significante entre regiões. Para acessibilidade geográfica, 60% dos usuários declararam que a unidade básica de saúde não ficava longe de sua residência, 83% afirmaram ser muito fácil/fácil chegar até a unidade e a maioria dos usuários relatou caminhar (64,5%). Para adequação, a unidade foi avaliada como muito bom/bom para os itens conforto (74,2%) e limpeza (90,9%), e 70,8% dos usuários relataram não ter de esperar para retirar seus medicamentos, embora o tempo médio de espera tenha sido 32,9 minutos. Para aceitabilidade: 93,1% dos usuários relataram ser atendidos com respeito e cortesia pelos funcionários das unidades dispensadoras e 90,5% declararam ser muito bom/bom o atendimento das unidades. Para capacidade aquisitiva 13% dos usuários relataram ter deixado de comprar algo importante para cobrir gastos com problemas de saúde, 41,8% dos participantes apontaram a despesa com medicamentos.

**CONCLUSÕES:**

Os resultados mostram 70%–90% de conformidade, compatível com países desenvolvidos. No entanto, o acesso aos medicamentos continua sendo um desafio pois ainda é fortemente comprometido pela baixa disponibilidade de medicamentos essenciais em unidades públicas de saúde, demonstrando que não ocorre de forma universal, equânime e resolutiva à população.

## INTRODUCTION

Access to medicines is an indispensable component for populations to have a universal and equal health coverage, with problem-solving capacity and quality, being recognized by the United Nations as one of the five indicators related to advances in ensuring the right to health^9^.

Brazil has adopted strategies aiming to expand access to safe and effective medicines, by regulations of great importance to the health system. The legal framework to ensure access to medicines has been established with the law 8080/1990^5^, which established the right of all citizens to integral therapeutic care, including pharmaceutical services. However, after numerous cases of quality deviations, forgeries, and inefficient sanitary control, a National Medicines Policy was elaborated and published in 1998, to ensure the access to essential medicines. This policy established the adoption and implementation of guidelines and priorities for government action, consisting in the reorientation of Pharmaceutical Services (PS), adoption of the *Relação Nacional de Medicamentos Essenciais* (RENAME – National List of Essential Medicines) and other items^11^. In 2004, the National Health Council also reaffirmed, by the *Política Nacional de Assistência Farmacêutica* (PNAF – National Policy of Pharmaceutical Services), the need for the Brazilian Unified Health System (SUS) to adopt actions for the expansion of access to medicines, development and local production of supplies and medicines according to the Brazilian needs, promotion of rational use, and qualification of the health professionals involved with medicines^12^.

Ensuring access to medicines is particularly important in the context of Primary Health Care (PHC), which is characterized as an entry in SUS, and it is part of the process of promotion, recovery, and prevention of some of the most prevalent diseases in the population.

In 2015, Brazil had more than 40 thousand basic health units (UBS) in operation, with a potential coverage of about 70% of the Brazilian population^a^. Therefore, the evaluation of access to medicines is essentially important, since a large portion of the Brazilian population, mainly that with lower income, relies on public programs and, in particular, on medicines offered by the Basic Component of SUS Pharmaceutical Services^8^.

Access to medicines depends on a complex network of public and private actors, who play different roles depending on the economic, political, and social context of several countries which encourage conditions for this access to happen^14^, and these countries must work together and gather political, social, and multidisciplinary efforts toward solutions.

In the academic field, the term “health services accessibility” presents a striking multiplicity of concepts and approaches. Conceptually, “health accessibility” has been used to represent different dimensions over time. The first proposals mapped by the World Health Organization in the 1970s suggested a strong connection of access with geographical accessibility, availability, and affordability. Latest literature seeks to address less tangible aspects, such as cultural, educational, and socioeconomic ones, incorporating the conceptual dimension of acceptability in the analyses^20^.

The development of a measuring instrument of access that considers the specificities of various health systems, as well as the context in which it is located, is a great challenge, because of the difficulty of measurement and variations of the health systems^7^. Penchansky and Thomas^17^ (1981) have defined access as the “degree of fit between clients and system,” and highlighted that a full analysis of access must include attributes of patients’ and health services’ needs. This analysis comprises a multidimensional concept, covering specific dimensions that include: availability, accessibility, accommodation, affordability, and acceptability.

Penchansky and Thomas^17^ based themselves on the observation of the pharmaceutical services model of North America and Western Europe, in which medicines were obtained in private community pharmacies, with or without funding by a third actor (public programs or private insurance). Despite differences between logistic models of pharmaceutical services, the observations and dimensions used by these authors are useful and have been used to evaluate and characterize access to medicines in different countries.

It has been estimated that, at the beginning of the 21^st^ century, one out of three people in the world would not have access to essential medicines, and, in low and middle income countries, this proportion could reach 50%^16^ ^,^ ^23^. In Brazil, population data on access to medicines are rare and often restricted to the provision of specific services and medicines^4^. National studies that evaluate access to medicines in the public sector^4^ ^,^ ^14^ ^,^ ^18^ have predominantly analyzed the dimension of availability, also observing absence of standardization in measures and other indicators of PS evaluation.

This study aimed to verify the access to medicines within the PHC of SUS, from the perspective of the patient, employing the multidimensional concept of access established by Penchansky and Thomas^17^.

## METHODS

PNAUM is a cross-sectional, exploratory, evaluative study, consisting of an information gathering in a representative sample of primary health care services in Brazilian cities. Several study populations were considered in the sampling plan, with samples stratified by regions, which constitute the study domains^1^. In-person interviews were held with patients, doctors, and those responsible for delivering medicines in SUS primary health care services, in addition to observation of the pharmaceutical services facilities and telephone interviews with administrators responsible for pharmaceutical services in the cities.

For randomly selecting the patients’ sample, we used sampling in three stages: city, health service, and patient. The organizers defined that 1,800 patients would be interviewed by region of the country. Considering the occurrence of a non-response percentage of 15%, 2,100 patients were randomly selected. In each region, this number of patients was proportionally distributed by the strata (city and health service), according to the frequency of services sampled in each of them. Patients were addressed within health services, while waiting for a medical appointment. The selection of patients in each service cannot be performed from patients’ listings. Thus, criteria for the selection of patients were established, making the selection be as close as possible to a random selection. Data were collected between July and December 2014.

The evaluation of access to medicines was conducted by the data obtained from interviews with patients, based on the five dimensions of access: availability, accessibility, accommodation, acceptability, and affordability. Each dimension was evaluated by its own indicators, as Table 1 shows.

**Table 1:** Consolidated indicators of access to medicines in the primary health care of SUS in Brazil, according to patients’ perception. National Survey on Access, Use and Promotion of Rational Use of Medicines – Services, 2015.

Access Dimensions	Concept^*^	Indicator	n	% (95%CI)
Availability	Relationship between the type of services and volume of existing resources according to the needs and volume of patients.	A1. % of patients who reported full access	3,357	59.8 (55.1–64.4)
		A2. % of patients who reported partial access	2,144	35.9 (31.7–40.3)
		A3. % of patients who reported no access	257	4.3 (3.0–6.1)
Accessibility	Relationship between location of the service and location of patients, considering resources of users of transportation, travel time, distance, and cost.	B1. % of patients who declared the UBS was far from their house	1,835	24.5 (22.2–27.0)
		B2. % of patients who declared it is very easy/easy getting to the UBS	7,204	83.0 (80.5–85.2)
		B3. % of patients who declared walking to the UBS	5,723	64.5 (60.9–67.9)
		B4. % of patients who rated as very easy/easy the existing signaling to find the dispensing unit of SUS	5,063	91.3 (88.8–93.2)
Accommodation	Represents the relationship between the way the services are organized to receive patients and the ability of patients to adapt to this organization.	C1. % of patients who declared the comfort of the SUS dispensing unit is very good/good	4,053	74.2 (68.0–79.5)
		C2. % of patients who declared the cleanliness of the SUS dispensing unit is very good/good	4,946	90.9 (87.6–93.4)
		C3. Average waiting time in minutes to receive medicines	1,628	32.93 (14.6–51.3)
		C4. % of patients who declared not waiting to withdraw medicines	4,082	70.8 (65.6–75.4)
		C5. % of patients who declared the opening hours of the UBS is very good/good	7,104	85.7 (83.7–87.5)
Acceptability	Represents the attitudes of individuals and providers regarding the characteristics and practices of each one.	D1. % of patients who declared to be always/repeatedly served with respect and courtesy	5,207	93.1 (91,8–94.2)
		D2. % of patients who declared the care performed by the SUS dispensing unit is very good/good	5,039	90.5 (88.7–92.0)
		D3. % of patients who declared the privacy in the care performed by the SUS dispensing unit where they receive medicines is very good/good	3,660	66.4 (61.2–71.0)
Affordability	Relationship between the cost of services and payment capacity of the patient or client.	E1. % of patients who reported the family was not able to buy something important to cover expenses with a health problem	937	13.0 (10.2–16.4)
		E2. % of patients who declared that medicines were the problem that caused this expense	389	41.8 (33.7–50.0)

SUS: Brazilian Unified Health System; UBS: basic health unit.

^*^ Concepts adapted from Penchansky and Thomas (1981).

Source: PNAUM – Services, 2015.

The evaluation of the “availability” dimension was made by the question: “In the last three months, how often did you get the medicines that you sought in SUS dispensing units?”, and the variable was categorized into full access (always), partial access (repeatedly, sometimes, or rarely), and no access (never).

“Accessibility” was evaluated by asking patients about how far and how easy/very easy it was for them to get to the UBS, whether it was possible to go walking, and about the existing signaling to find the dispensing unit in the UBS.

“Accommodation” was observed by the patients’ perceptions regarding comfort, cleanliness, waiting time, and opening hours of the UBS.

“Acceptability” was analyzed by the patients’ perception about the quality of service, specifically concerning courtesy, respect, and privacy in the care.

“Affordability” was examined by asking patients if their family stopped buying something important to cover health expenses, and whether these expenses were related to the purchase of medicines.

Data were analyzed using the software SPSS®, version 22. All analyses considered the sampling weights and structure of the complex plan. The results show representativeness for the geographic regions of Brazil. Tables of distribution of frequencies for categorical variables and of measures of central tendency for numerical variables were built. To evaluate the statistical association, Student’s t test was conducted for numeric variables, and Pearson correlation test was held for categorical variables. The significance level adopted was p < 0.05.

PNAUM was approved by the Research Ethics Committee, by Opinion no. 18947013.6.0000.0008. All participants signed the informed consent form.

## RESULTS

Of the 8,803 patients interviewed in the UBS of the five regions of Brazil, 8,591 (97.5%) answered to the questionnaire items related to the dimensions “accessibility” and “affordability,” interpreted as stopping to buy something important to cover health expenses. Only patients who have used/searched medicines in the SUS dispensing units (65.4%, n = 5,758) answered to the items about the other dimensions evaluated.

Table 1 presents the consolidation of the indicators on the various dimensions of access in Brazil. Table 2 shows a detailed analysis by Brazilian region of the patients’ perception about access to medicines in SUS Primary Health Care, classified by dimension.

**Table 2:** Patients’ perception on access to medicines in primary health care of SUS, classified by dimension and region of Brazil. National Survey on Access, Use and Promotion of Rational Use of Medicines – Services, 2015.

	North		Northeast		Midwest		Southeast		South	
				
	n^a^	% (95%CI)^b^	n^a^	% (95%CI)^b^	n^a^	% (95%CI)^b^	n^a^	% (95%CI)^b^	n^a^	% (95%CI)^b^
A1. Perception on the access to medicines in SUS										p = 0.167
Total access	561	54.2 (43.6–64.5)	515	57.0 (49.3–64.3)	473	46.3 (40.0–52.4)	777	64.3 (53.4–73.9)	1,031	60.8 (54.2–67.0)
Partial access	407	39.1 (30.7–48.2)	427	36.8 (29.6–44.6)	399	46.6 (40.6–53.0)	468	32.5 (24.0–42.5)	443	36.4 (30.8–42.5)
No access	67	6.7 (3.2–13.2)	52	6.3 (3.1–12.2)	70	7.1 (4.7–10.8)	38	3.2 (2.0–5.1)	30	2.8 (1.6–4.8)
B1. Accessibility – Is the UBS far from the patients’ house?										p = 0.813
Yes	280	20.2 (15.9–25.3)	478	29.6 (23.8–36.1)	314	22.0 (17.8–27.0)	346	21.6 (18.5–25.0)	417	23.9 (20.4–27.8)
More or less	245	16.4 (12.7–20.8)	284	15.6 (12.9–18.7)	247	16.4 (13.0–20.4)	288	16.3 (13.0–20.6)	297	15.7 (12.3–19.8)
No	1,021	63.4 (56.3–70.0)	919	54.8 (47.1–62.3)	953	61.5 (55.6–67.0)	1,196	62.1 (57.0–66.8)	1,306	60.4 (55.5–65.2)
B2. Accessibility – Facility to go to the UBS										p = 0.358
Very easy/easy	1,277	81.5 (77.0–85.3)	1,373	81.9 (76.0–86.7)	1,255	80.3 (76.0–83.8)	1,568	84.6 (80.2–88.0)	1,731	82.9 (79.1–86.2)
Neither easy nor difficult	160	10.5 (8.0–13.8)	158	9.0 (6.4–12.5)	137	11.2 (8.6–14.5)	149	9.4 (7.2–12.1)	139	7.6 (5.9–9.8)
Difficult/very difficult	109	7.9 (5.4–11.5)	150	9.0 (6.3–12.8)	122	8.5 (6.6–10.9)	114	6.1 (4.0–9.2)	149	9.4 (7.2–12.2)
B3. Accessibility – Patients’ means of transport to get to the UBS										p < 0.05
Walking	1,039	60.4 (50.8–69.3)	1,161	66.2 (57.6–74.0)	859	52.6 (48.0–52.6)	1,349	70.1 (64.0–75.6)	1,315	58.4 (53.4–63.3)
Bus^c^	75	3.3 (2.3–4.8)	152	4.2 (2.8–6.4)	77	3.5 (1.9–6.2)	165	5.7 (4.3–7.4)	157	7.1 (4.7–10.6)
Car/Motorcycle	341	22.2 (15.7–30.4)	349	27.8 (21.4–35.0)	461	32.8 (28.5–37.0)	355	23.3 (17.5–30.0)	577	34.5 (30.3–38.9)
Boat/Other	123	8.1 (5.7–11.5)	64	4.7 (3.0–7.2)	123	9.5 (7.0–12.8)	37	2.5 (1.5–4.2)	59	3.8 (2.1–6.6)
B4. Accessibility – Existing signaling in the UBS to find the SUS dispensing units										p = 0.024
Very easy/easy	880	86.9 (81.4–90.9)	858	93.3 (90.5–95.3)	820	89.1 (86.0–91.6)	1,143	92.6 (87.6–95.7)	1,362	88.9 (83.3–92.8)
Neither easy nor difficult	96	9.3 (6.2–13.6)	93	5.0 (3.7–6.6)	45	5.5 (3.1–9.6)	70	5.3 (3.1–8.8)	58	4.8 (2.7–8.2)
Difficult/very difficult	48	3.9 (2.3–6.4)	20	1.7 (0.7–4.1)	59	5.4 (3.4–8.3)	41	2.1 (1.1–4.2)	69	6.3 (3.6–11.1)
C1. Accommodation – Comfort of the SUS dispensing unit where the patient receives medicines										p = 0.025
Very good/good	661	64.2 (54.5–72.8)	581	66.7 (52.3–78.5)	685	74.9 (65.9–82.2)	975	82.2 (74.7–87.8)	1,151	73.0 (59.5–83.3)
Neither good nor bad	227	22.2 (16.3–29.4)	183	12.0 (7.9–17.8)	151	15.1 (10.8–20.7)	167	11.3 (8.4–15.0)	188	14.3 (10.2–19.6)
Bad/very bad	143	13.7 (9.5–19.2)	215	21.4 (13.5–32.1)	93	10.0 (6.2–15.7)	130	6.5 (3.6–11.6)	156	12.7 (6.6–22.8)
C2. Accommodation – Cleanliness of the SUS dispensing unit where the patient receives medicines										p = 0.249
Very good/Good	823	82.8 (75.6–88.2)	786	88.3 (78.8–93.9)	802	91.7 (85.1–95.5)	1,161	93.0 (86.1–96.6)	1,374	92.3 (88.2–95.0)
Neither good nor bad	142	14.0 (9.8–19.6)	114	9.5 (4.7–18.3)	59	6.2 (3.6–10.4)	66	5.1 (2.7–9.3)	74	5.7 (3.8–8.4)
Bad/very bad	31	3.2 (1.7–5.8)	28	2.2 (1.1–4.1)	19	2.1 (0.8–5.3)	23	1.9 (0.7–5.2)	24	2.1 (1.0–4.1)
C3. Accommodation – Waiting time in minutes to receive medicines in SUS										p = 0.173
Average	154	13.3 (10.8–15.7)	242	18.2 (11.0–25.1)	179	14.5 (11.6–17.0)	508	59.1 (11.0–107.0)	545	18.1 (11.0–25.1)
C4. Accommodation – Perception of time spent to receive medicines in SUS units										p = 0.008
No time	879	85.6 (78.7–90.5)	738	80.1 (71.6–86.6)	749	77.8 (69.3–84.5)	763	69.2 (57.9–78.5)	953	59.2 (52.6–65.6)
A little	133	12.7 (8.7–18.2)	204	17.9 (12.0–25.8)	151	18.9 (12.7–27.1)	413	24.0 (18.7–30.2)	453	34.3 (26.0–43.6)
A long time	21	1.8 (0.7–4.5)	38	2.0 (0.9–4.4)	28	3.3 (1.8–6.1)	95	6.8 (2.9–15.2)	92	6.6 (2.6–15.7)
C5. Accommodation – Evaluation of the opening hours of the UBS										p = 0.001
Very good/good	1,242	81.8 (76.8–86.0)	1,267	80.5 (74.9–85.1)	1.259	84.8 (79.9–88.7)	1,578	89.8 (87.3–91.8)	1,758	87.5 (84.1–90.2)
Neither good nor bad	203	13.2 (9.8–17.6)	260	12.6 (9.4–16.6)	170	10.8 (7.9–14.6)	165	7.6 (6.2–9.4)	168	8.2 (6.2–10.7)
Bad/very bad	78	4.9 (3.6–6.8)	124	6.9 (5.1–9.3)	77	4.4 (3.1–6.1)	59	2.6 (1.7–3.9)	80	4.3 (2.5–7.4)
D1. Acceptability – Do the staff of the SUS dispensing units attend patients with respect and courtesy?										p = 0.175
Always/repeatedly	921	91.0 (89.4–93.8)	874	91.8 (89.2–93.8)	839	91.9 (89.4–93.8)	1,174	94.8 (93–96.2)	1,399	93.0 (89.9–95.2)
Sometimes	92	7.3 (5.1–10.3)	88	6.7 (4.5–9.9)	74	6.4 (4.6–8.9)	76	3.6 (2.6–5.1)	79	5.4 (3.8–7.5)
Rarely/never	22	1.7 (1.0–2.9)	25	1.5 (0.8–2.7)	23	1.7 (1.1–2.8)	27	1.6 (1.0–2.5)	22	1.6 (0.9–3.2)
D2. Acceptability – Evaluation of the service performed by the SUS dispensing units										p = 0.046
Very good/good	872	85.2 (79.9–89.2)	848	90.6 (88.4–92.4)	806	88.1 (83.7–91.5)	1,161	93.2 (90.7–95.0)	1,352	88.5 (83.5–92.1)
Neither good nor bad	130	12.2 (8.7–16.9)	105	7.4 (6.1–9.0)	95	9.0 (6.3–12.6)	88	5.2 (4.0–6.9)	116	8.9 (6.1–12.7)
Bad/very bad	28	2.6 (1.4–4.9)	33	2.0 (1.3–3.3)	33	2.9 (1.6–5.3)	27	1.6 (0.8–3.2)	31	2.7 (1.6–4.2)
D3. Acceptability – Evaluation of the privacy in the service performed by the SUS dispensing units										p = 0.010
Always/repeatedly	714	69.2 (48.5–64.8)	551	61.8 (52.8–70.0)	520	56.8 (48.5–65.0)	827	73.1 (62–81.6)	1,048	63.7 (56.8–70.0)
Sometimes	131	12 (8.0–17.5)	176	13.8 (10.9–17.0)	165	16.7 (11.8–23.0)	136	11.1 (7.3–16.4)	164	12.6 (9.8–16.0)
Rarely/never	182	18 (9.8–30.7)	168	15.7 (10.0–23.8)	235	24.4 (16.0–35.2)	265	12.8 (8.4–19.0)	263	21.3 (15.4–28.6)
E1. Affordability – Were you not able to buy something important to cover expenses for any health problem?										p = 0.533
Yes	123	7.5 (5.4–10.4)	224	14.7 (10.3–20.6)	148	9.3 (5.4–15.5)	194	12.1 (6.6–21.0)	248	14.5 (9.8–20.8)
No	1,420	92.5 (89.6–94.6)	1,454	85.3 (79.4–89.7)	1,368	90.7 (84.5–94.6)	1,633	87.9 (79.0–93.0)	1,771	85.5 (79.2–90.2)
E 1.2. Affordability – % of patients who declared that medicines were the problem that caused this expense										p = 0.429
Yes	40	27.3 (20.1–36.0)	101	45.6 (35.0–56.7)	67	42.4 (30.0–55.9)	87	45.8 (25.7–67.0)	94	34.1 (25.9–43.0)

SUS: Brazilian Unified Health System; UBS: Basic Health Unit.

^a^ not weighted; ^b^ % weighted; ^c^ to bus or public transport, the p-value was > 0.05.

Source: PNAUM – Services, 2015.

In “availability” dimension, regarding the item “access to medicines in SUS dispensing units,” it was found that 59.8% of patients reported full access to medicines in SUS, which was higher in the Southeast (64.3%) and lower in the Midwest (46.3%). “Partial access” and “no access” to medicines in the SUS corresponded respectively to 35.9% and 4.3% of the patients interviewed. Statistically significant differences were not observed between regions of the Country (p = 0.167).

In “accessibility” dimension, 59.5% of patients declared that the “UBS is not far from their house,” and most patients (83.0%) considered that it is easy or very easy to get to the unit, with about 64.5% of them going to the health unit walking. Still regarding the means of transport used to go to the units, a higher proportion of patients in the Southeast region reported that they go walking (70.0%), while in the Midwest this proportion was lower (52.6%). The South region presented higher frequency of patients who use car or motorcycle (34.5%) and the Northern region, the lowest (22.2%). Statistically significant differences were found between the regions (p < 0.05). Concerning the evaluation of the existing signaling, most patients (88.5%) declared it was very easy/easy to find the SUS dispensing unit.

In the “accommodation” dimension, the comfort and cleanliness of the SUS dispensing units were evaluated as very good/good by 74.2% and 90.9% of patients, respectively. For comfort, statistically significant differences were observed between regions (p < 0.05); in the Southeast region, 82.2% of patients evaluated SUS units as very good, and in the Northeast, 64.2% and 66.7%, respectively.

The average waiting time to receive medicines in SUS dispensing units was 32.9 minutes (min), with the longest time reported in the Southeast region (59.8 min) and the shortest, in the North (13.3 min). Nevertheless, 70.1% of patients reported not waiting to receive their medicines.

It was found that 85.8% of patients evaluated the opening hours of the UBS as very good/good, with the highest proportion in the Southeast region (89.8%) and the lowest in the Northeast (80.5%), showing statistically significant differences between regions (p < 0.05).

In “acceptability” dimension, 93.1% of patients reported to be served with respect and courtesy by the staff of the SUS dispensing units. The service was well evaluated: 90.5% of patients declared the service of these units was very good/good; but patients of the North region (85.2%) were least satisfied with it (p < 0.05). Regarding privacy in the care, a percentage of satisfaction of 66.4% was observed, with 73.1% in the Southeast region and 56.8% in the Midwest (p < 0.05).

In “affordability” dimension, 13% of patients in Brazil reported not being able to buy something important to cover expenses with a health problem, and 41.8% of them declared that it was to buy medicines. Statistically significant differences were not found between the regions.

## DISCUSSION

The multidimensional evaluation of access to medicines according to the patients’ perspective is vitally important to identify aspects and factors that go beyond the simple provision of medicines. The perspective of the five dimensions, adapted from Penchansky and Thomas^17^ (1981), of this study allowed us to know the perception of patients, who are the main beneficiaries of SUS and to whom health policies must be aimed at^10^.

“Availability” dimension is still recognized as the main obstacle to access in Brazil. It is a problem that persists in the Country, and several studies carried out in the public sector have found problems with physical availability, acquisition, or lack of medicines^1^ ^-^ ^3^ ^,^ ^6^ ^,^ ^15^ ^,^ ^18^ ^,^ ^19^. In this study, we observed low levels of availability of medicines in the PHC (46.3% to 64.3%) among patients who have declared to have full access to medicines by the SUS dispensing units, which may impair the integrality of health care.

The highest frequency of patients who reported full access was in the Southeast, and the smallest, in the Midwest, confirming the findings of Boeing et al.^4^ (2013) in the National Household Sample Survey of 2008. In this study, 45.3% of individuals reported having had full access (received all prescribed medicines by SUS), but with a higher proportion in the South region (48.1%) and a lower one in the North (37%).

The accessibility to dispensing units in the primary health care presents some conflicting results, according to the patients’ perspective. Although most patients declared it was easy or very easy to reach the UBS, nearly a quarter of patients reported that the UBS was far from their house. It is worth mentioning that the organization by registered population, i.e., the population of the service area of a health unit, adopted in PHC, should minimize the problems of “accessibility.” The PHC aims to ensure citizens an ordered and organized access to health systems, primarily by services of PHC found close to the houses of patients, to guarantee the integrality of the health care^22^.

A higher proportion of patients in the Southeast region declares that they go walking to the UBS, while patients of the South used car or motorcycle (p < 0.05). A study conducted with 188 patients of six UBSs in a city of Minas Gerais shows that 89.4% of patients declared to be satisfied regarding location, distance from the house, and time spent to reach the unit, besides the possibility of not needing to use means of transport^21^. In another study conducted in two UBSs of Recife, 76% of patients (n = 1,161) were satisfied with the distance from their house to the UBS^7^. Concerning the quality of the signaling of the SUS medicine dispensing units, 91.3% of patients considered it was easy/very easy. However, we found no other similar studies for comparison.

For the “accommodation” dimension, most patients of this study considered the UBSs clean and comfortable. Patients in the Southeast, although being pleased with the comfort, cleanliness, and opening hours, reported having to wait about one hour on average to withdraw the medicines. A study held in a city of Minas Gerais^6^ found that the average waiting time in line at the pharmacy was three minutes, a much lower value than the one found in this research.

For “acceptability” dimension, patients considered the service as good/very good and evaluated the treatment by the staff as respectful and polite. Patients in the Southeast region were the most satisfied, and those of the North, the least. Regarding privacy in the service, 15% of patients reported rarely/never having privacy, which was more common in the Midwest region. A systematic review by Nora and Junges^13^ pointed out that the lack of appropriate physical space in the UBSs may be related to the absence of privacy in the care to patients. They also reported that the discontent of patients with the physical space, considered small, or even the lack of a waiting room, makes the waiting for care an uncomfortable experience. In this review, the comfort in the dispensing units of medicines was considered unsatisfactory.

For the “affordability” dimension, 13.0% of patients reported that they were not able to buy something important to cover health expenses. This result must be carefully seen, since patients with access problems may not be present in the health services at the time of the interview, resulting in less representation. Thus, the obtained indicator may be underestimating the extension of the economic impacts for the families due to the low availability of medicines in SUS^17^. Despite this possible bias, medicine was considered one of the main problems that caused health spending. The same was observed in a study on Household Budget Surveys in the years 2002-2003 and 2008-2009, by the Brazilian Institute of Geography and Statistics, which found that the expenses on medicines is the main component of the health expenditures of Brazilian families. This affected more the families with less income, which clearly separated, proportionally, a greater share of family income in the acquisition of medicines^8^.

Evaluating access to medicines in Brazil is still a major challenge, especially regarding the multiplicity of concepts and approaches on the subject. In addition to impairing the comparability of the studies, the lack of standardization between the instruments used in the evaluations causes the indicators to show discrepant results between dimensions. Results of this study on access to medicines are representative of Brazil. Evaluating the different dimensions from the perspective of patients of SUS and the consequent performance of public policies to this end provides data with implications on the management and allocation of resources in the health sector.

The results, from the patients’ perspective, to the dimensions of access (accessibility, accommodation, acceptability, affordability) are compatible with developed countries, with indicators situated between 70% and 90% compliance. However, access to medicines is still heavily compromised by the low availability of essential medicines in public health units, confirming that it does not occur universally, equally, and decisively to the population, and that it remains a challenge to SUS.

## Footnotes

a SAGE - Sala de Apoio à Gestão Estratégica. Unidade Básica de Saúde. Brasília (DF): Ministério da Saúde; 2015 [cited 2015 Mar 23]. Available from: http://sage.saude.gov.br/.

## References

[1] Álvares J, Alves MCGP, Escuder MML, Almeida AM, Izidoro JB, Guerra AA (2017). Pesquisa Nacional sobre Acesso, Utilização e Promoção do Uso Racional de Medicamentos: métodos. Rev Saude Publica.

[2] Arakawa T, Arcêncio RA, Scatolin BE, Scatena LM, Ruffino-Netto A, Villa TCS (2011). Accessibility to tuberculosis treatment: assessment of health service performance. Rev Lat Am Enfermagem.

[3] Bertoldi AD, Helfer AP, Camargo AL, Tavares NUL, Karanavos P (2010). Medicine prices, availability and affordability in Southern Brazil: a study of public and private facilities.

[4] Boing AC, Bertoldi AD, Boing AF, Bastos JL, Peres KG (2013). Acesso a medicamentos no setor público: análise de usuários do Sistema Único de Saúde no Brasil. Cad Saude Publica.

[5] Brasil (1990). Lei nº 8.080 de 19 de setembro de 1990. Dispõe sobre as condições para a promoção, a organização e o funcionamento dos serviços correspondentes e dá outras providências. Diario Oficial Uniao.

[6] Chaves GC, Emmerick I, Pouvourville N, Saint-Denis T, Fonseca ASA, Luiza VL (2005). Indicadores de uso racional de medicamentos e acesso a medicamentos: um estudo de caso. Rev Bras Farm.

[7] Emmerick ICM (2011). Dimensões e determinantes do acesso a medicamentos em três países da América Central.

[8] Garcia LP, Magalhães LCG, Sant’Anna AC, Freitas LRS, Aurea AP (2013). Dimensões do acesso a medicamentos no Brasil: perfil e desigualdades dos gastos das famílias, segundo as pesquisas de orçamentos familiares 2002-2003 e 2008-2009.

[9] Hogerzeil HV, Mirza Z (2011). The world medicines situation 2011: access to essential medicines as part of the right to health.

[10] Mendes ACG, Miranda GMD, Figueiredo KEG, Duarte PO, Furtado BMASM (2012). Acessibilidade aos serviços básicos de saúde: um caminho ainda a percorrer. Cienc Saude Coletiva.

[11] Ministério da Saúde (BR) (1998). Portaria nº 3.916 de 30 de outubro de 1998. Aprova a Política Nacional de Medicamentos. Diario Oficial Uniao.

[12] Ministério da Saúde (BR), Conselho Nacional de Saúde (2004). Resolução nº 338, de 06 de maio de 2004. Aprova a Política Nacional de Assistência Farmacêutica.

[13] Nora CRD, Junges JR (2013). Política de humanização na atenção básica: revisão sistemática. Rev Saude Publica.

[14] Organização Pan-Americana da Saúde (2005). Avaliação da Assistência Farmacêutica no Brasil: estrutura, processo e resultados.

[15] Paniz VMV, Fassa ACG, Facchini LA, Bertoldi AD, Piccini RX, Tomasi E (2008). Acesso a medicamentos de uso contínuo em adultos e idosos nas regiões Sul e Nordeste do Brasil. Cad Saude Publica.

[16] Paniz VMV, Fassa ACG, Facchini LA, Piccini RX, Tomasi E, Thumé E (2010). Free access to hypertension and diabetes medicines among the elderly: a reality yet to be constructed. Cad Saude Publica.

[17] Penchansky R, Thomas JW (1981). The concept of access: definition and relationship to consumer satisfaction. Med Care.

[18] Pinto CBS, Miranda ES, Emmerick ICM, Costa NR, Castro CGSO (2010). Preços e disponibilidade de medicamentos no Programa Farmácia Popular do Brasil. Rev. Saude Publica.

[19] Rodrigues AMS, Scatena LM, Vendramini SHF, Canini SRMS, Villa TCS, El Gir (2012). Avaliação do acesso ao tratamento de tuberculose por coinfectados ou não pelo vírus da imunodeficiência humana. Rev Esc Enferm USP.

[20] Sanchez RM, Ciconelli RM (2012). Conceitos de acesso à saúde. Rev Panam Salud Publica.

[21] Santos SMS, Oliveira VAC, Oliveira RAC, Guimarães EAA (2010). Estratégia Saúde da Família: qualidade da assistência sob a perspectiva do usuário. REME Rev Min Enferm.

[22] Starfield B (2002). Atenção primária: equilíbrio entre necessidades de saúde, serviços e tecnologia.

[23] World Health Organization (2000). WHO medicines strategy: framework for action in essential drugs and medicines policy 2000-2003.

